# Understanding Beech Predominance Through Sapling and Adult Demography Along Environmental Gradients

**DOI:** 10.1002/ece3.74399

**Published:** 2026-09-22

**Authors:** Lukas Heiland, Georges Kunstler, Lisa Hülsmann

**Affiliations:** ^1^ University of Bayreuth, Bayreuth Center of Ecology and Environmental Research (BayCEER), Ecosystem Analysis and Simulation (EASI) Lab Bayreuth Germany; ^2^ Theoretical Ecology, Universität Regensburg, Universitätsstraße 31 Regensburg Germany; ^3^ Université Grenoble Alpes, Inrae, LESSEM Grenoble France

**Keywords:** Bayesian inverse calibration, dynamic species distribution model, ecogram, *Fagus sylvatica*, German national forest inventory, plant community dynamics

## Abstract

Understanding how species abundances are driven by biotic interactions along environmental gradients is a fundamental ecological question. In Central European forests, a classical theory by Ellenberg predicts that beech ( *Fagus sylvatica* L.) outcompetes other tree species within a mesic range of soil pH and water levels, while other species prevail under less favorable conditions. While the theory is generally accepted in ecology, only certain aspects of it have been substantiated by empirical evidence. Moreover, the demographic drivers of the turnover from beech to other tree species along the soil gradients remain unexplained. To address this, we inversely calibrated a parsimonious forest model with a sapling stage and interacting populations using short time series of tree abundances from the German national forest inventory. By modeling how demographic rates for beech and the other species—grouped into an average species—vary along pH and soil water gradients in a disturbed system, we tested the prediction that beech predominates only under favorable soil conditions at equilibrium. Moreover, we tested with simulations how environmentally responsive demographic rates explain beech's changing abundance along the two gradients. Our results largely confirm that beech outcompetes the grouped other species in a central environmental range. Change of beech's relative abundance along environmental gradients is primarily explained by variation in its net basal area increment, followed by its competition response at both overstory and sapling stage. Although sapling tolerance to shading is the primary mechanism for beech predominance, its variability is secondary for change in its relative abundance along environmental gradients. For the first time, we substantiate Ellenberg's theory by elucidating how species turnover along environmental gradients is based in demography. Our approach of calibrating demographic rates that respond to the environment can be utilized to predict distributions of interacting species and explain their dynamics across environmental gradients.

## Graphical abstract

We address the question how species abundances are driven by biotic interactions along environmental gradients by applying a demographic population model to NFI data, substantiating a classical ecological theory by Ellenberg: The dominant species in Central European forests outcompetes other tree species only under favorable environmental conditions. Our novel approach demonstrates how species abundances are driven by demographic processes and species interactions, depending on the environment.

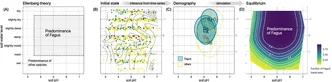

## 1 Introduction

Understanding where species occur is one of the central questions in ecology (MacArthur 1972; Sutherland et al. 2013). Ecologists have used the Hutchinson niche concept (Hutchinson 1957) and abundance response curves (Ellenberg 1952; Whittaker 1967; Ter Braak and Prentice 1988) to describe changes in species abundance along environmental gradients. In the quest to go beyond description, ecologists since Maguire (1973) have sought to explain changes in abundance with environmentally‐dependent demographic processes (Watkinson 1985; Schurr et al. 2012; Normand et al. 2014). One approach involves dynamic species distribution models (SDMs) that link demographic rates to environmental conditions (e.g., Higgins et al. 2012; Schurr et al. 2012; Normand et al. 2014; Pironon et al. 2017; Kunstler et al. 2021; Schultz et al. 2022). Here, we use a dynamic SDM for size‐structured communities, to explain species' turnover based on how the underlying demographic rates change along environmental gradients. Applied to data from disturbed forest systems, we use the inferred demographic rates to simulate equilibrium conditions and test Ellenberg's (1963) classical theory on the predominance of *Fagus sylvatica* in Central European forests.

Ellenberg's theory states that under equilibrium conditions, the late‐successional tree species *Fagus sylvatica* L. (common beech, hereafter *Fagus*) predominates at mesic soil conditions of submontane Central European forests, because under these conditions it is more competitive than other species (Ellenberg 1963). Mesic soil conditions—those without extreme chemical properties (indicated by intermediate to high pH; Härdtle et al. 2004) and without shortage or excess of water—are considered physiologically optimal for most tree species. Without species interactions, most tree species would therefore show a similar *physiological response* along soil pH and water gradients (Ellenberg 1952). At mesic soil conditions, *Fagus* is thought to outcompete other tree species, so that their realized, that is, *ecological*, optima are pushed away from their shared physiological optimum towards more extreme environments (Ellenberg 1963; Keddy 2001). *Fagus*' competitive demographics compared to other species include better shade‐tolerance of seedlings and saplings (Wagner et al. 2010; Käber et al. 2021), which creates a positive feedback mechanism between sapling recruitment and the shading from the overstory so that at late successional stages *Fagus* predominates on average (see also Peters 1997; Leuschner and Ellenberg 2017; Petrovska, Brang, et al. 2021; Heiland et al. 2023). Towards the extremes of the soil gradients, *Fagus* loses its dominance to other tree species, although the demographic processes underlying its reduced performance remain poorly understood.

Throughout forest ecology, the Ellenberg theory is generally accepted (e.g., Barna and Bosela 2015; Mellert et al. 2016; Leuschner 2020; Cailleret et al. 2020), partly because the predicted pattern of *Fagus*' predominance under mesic environmental conditions has been supported by numerous descriptions of natural forests (Peters 1997; Leuschner et al. 2006; Bolte et al. 2007; Axer et al. 2021; but see minor modifications by Leuschner and Ellenberg 2017). However, only some elements of the mechanisms postulated by Ellenberg (1963) have been evidenced. Specifically, studies have shown physiological constraints on *Fagus* in highly acidic soil conditions (Leuschner et al. 2006; Aertsen et al. 2012), but not in calcareous soils (Ljungström et al. 1990). Also in wet, waterlogged soils, *Fagus* experiences reduced physiological functioning (Dreyer 1994; Schmull and Thomas 2000; Scharnweber et al. 2013; Axer et al. 2021), leading to increased adult mortality (Gorzelak 2000). While *Fagus* is moderately drought‐tolerant (Niinemets and Valladares 2006; Rötzer et al. 2017; Leuschner 2020), extreme drought conditions make saplings susceptible to embolism (Tomasella et al. 2019), reduce nitrogen uptake (Fotelli et al. 2001; Geßler et al. 2006; Leuschner 2020), and inhibit adult growth (Ciais et al. 2005). A study on species interactions by Fichtner et al. (2012) highlighted that competition has a more severe effect on adult beech in less productive environments along soil chemistry and water level gradients and Jacobs et al. (2022) showed that the negative competition effect from *Fagus* on *Quercus petraea* (Matt.) Liebl. disappeared in dry conditions. The specific demographic drivers of species turnover along these soil gradients, however, remain unclear. *Fagus*' exceptional shade‐tolerance has been confirmed by several studies (Niinemets and Valladares 2006; Petritan et al. 2007; Käber et al. 2021; Petrovska, Brang, et al. 2021). In particular, Heiland et al. (2023) demonstrated with a size‐structured forest population model that saplings' shade‐tolerance is the key for *Fagus*' predominance at the competitive equilibrium. However, it has not been verified that *Fagus*' demographic rates vary across the environment in a way that allows it to outcompete other species only under mesic conditions. Moreover, the specific variation in demographic processes that drives species turnover towards the extremes of the soil gradients remains unclear (see generally Meier et al. 2011; Maréchaux et al. 2021). Therefore, here we ask how environmental variation in demographic rates of *Fagus* and the other competing tree species drives *Fagus*' relative abundance along environmental gradients?

**FIGURE 1:**
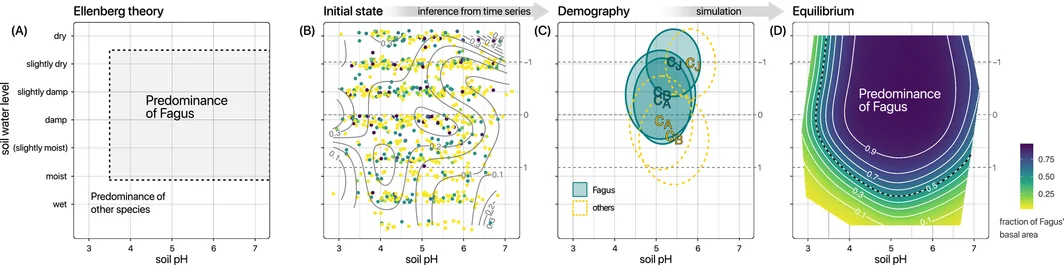
Hypothesis and modeling steps. The environmental range along the two axes pH and soil water level where *Fagus sylvatica* predominates, as postulated by Ellenberg (1963). (A) However, in disturbed forests plots of the first German NFI survey, *Fagus*' does not predominate, as indicated by the fraction of the total basal area at the initial subpopulation states (B; color scale as in panel D). As an example for the environmentally‐responsive demographic rates, specifically the high competitive tolerance of the size stages J, A, and B, the parameters $c_{J}$, $c_{A}$, and $c_{B}$ are represented by ellipses (C). The ellipses visualize the standard deviation around the location of the optima (center parameter κ). Variable competitive equilibria along environmental gradients (i.e., fraction of total basal area represented by *Fagus*); (D) were simulated with the fitted model. Points in panel B, representing forest plots in environmental space, were jittered by 0.1 in both directions.

To tackle this question, we used the JAB forest population model (Heiland et al. 2023). This parsimonious dynamic model explicitly includes demography of three size stages including the sapling stage and the competitive interactions between two populations, *Fagus sylvatica* and all *other* species. Its focus on competition within and between populations, especially from the overstory onto the sapling stage, makes it particularly well suited to address this question. We combined all non‐Fagus species into a single background population with environment‐dependent performance, to reduce computational cost. We fitted the model to short time series from the German NFI, using Bayesian inverse calibration to estimate population‐specific, environmentally‐responsive demographic rates (Hartig et al. 2012). This allows us to infer how demographic rates of *Fagus sylvatica* vary as a function of soil pH and water level (a two‐dimensional environmental space, that is, ecogram, according to Ellenberg 1963, see Figure A). The demographic rates of *others*, however, which are a species group and therefore not a population in the biological sense, can be interpreted as the response of an average population at the respective environments. With these population‐specific and environmentally‐responsive rates (Figure 1C), we simulated competitive equilibria to test the prediction that *Fagus sylvatica* will be the predominant population at mesic environmental conditions (Figure 1D). Further, we used a simulation method to test which demographic rates varies in response to the environment in a way that it explains the change in *Fagus*' relative abundance along the environmental gradients.

## 2 Materials and Methods

All data preparation and posterior analyses were performed with R (version 4.2.3; R Core Team 2021). The JAB model was implemented in stan (version 2.31.0; Stan Development Team 2022).

### 2.1 JAB Model

The JAB model (Heiland et al. 2023) is a simple dynamic forest population model that includes three size stages including the sapling stage. The model concentrates on competition between multiple populations and on their specific sapling dynamics by assuming a simple two‐layer size structure (see also Cordonnier et al. 2019).

The three stages of the JAB model include the juvenile stage J, representing the understory, and the stages A and B, jointly representing the overstory (Figure S1). The overstory stage (BA) is divided into the intermediary stage A and the final stage B, allowing conversion between count density in the sapling stage J and basal area as a measure for competition in the final stage B (Biging and Dobbertin 1992). The overstory (stages A and B) shades the understory, but is itself not affected by the understory J (Angelini et al. 2015; De Lombaerde et al. 2019), that is, asymmetric competition (Schwinning 1998).

The JAB model incorporates several key demographic processes that are population‐specific (here, we call these processes, including competition effects; Table 1). In the sapling stage J, these include the competition response to the overstory's basal area BA (s), competition among saplings ($c_{J}$), transition resulting from growth and survival to stage A (g), external seedling input ($L_{p}=B_{p}\;l$, see Section SA.4), and seedling regeneration from the local overstory (r). Transition from stage A to B is represented by the growth and survival rate h together with a conversion factor from counts to basal area ($\beta_{\mathrm{uA}}$, see Section SA.1). Basal area growth in the final stage B is captured by the net increment rate b. The overstory stages A and B have competition responses to BA ($c_{A}$ and $c_{B}$) so that all stages are logistically limited by the overall tree density through different competition parameters (J: s, $c_{J}$; A: $c_{A}$; and B: $c_{B}$). The density dependence applies not only to the stages but also to the growth‐related rates g, h, and b so that in addition to these rates at the absence of competition, we report estimates of the density‐dependent growth terms $\frac{g}{1+s\mathrm{sum}\left(\mathrm{BA}\right)+c_{J}\mathrm{sum}\left(J\right)}$, $\frac{h}{1+c_{A}\mathrm{sum}\left(\mathrm{BA}\right)}$, and $\frac{b}{1+c_{B}\mathrm{sum}\left(\mathrm{BA}\right)}$ (Table 2 and Figure S7).

**TABLE 1:** Demographic rates in the JAB model and their explanation.

Stage	Symbol	Shorthand	Explanation	Effect from …	On	(Direction)
J	$L_{p}$/l	Input	Seedling input from large‐scale and long‐term basal area average to specific J	B	J	+
	r	Recruitment	Seedling recruitment from the local basal area to specific J	BA	J	+
	$c_{J}$	Juvenile competition	Competition effect from total count of juveniles on specific J	Sum J	J	−
	s	Shading	Competition effect from total basal area on specific J	Sum BA	J	−
J/A	g	Transition to A	Transition due to survival and growth from specific J to specific A	J/A	A/J	+/−
A	$c_{A}$	Competition on A	Competition effect from total basal area on specific A	Sum BA	A	−
A/B	h	Transition to B	Survival and growth from specific A to specific B	A/B	B/A	+/−
B	b	Net basal area growth	Net basal area increment of specific B dependent on specific B (also includes mortality)	B	B	+
	$c_{B}$	Competition on B	Competition effect from all basal area on specific B (includes both mortality and growth reduction)	Sum BA	B	−

*Note:* The rates generally link two model states, so that there is an effect acting from some model state on another that can be positive or negative (indicated in column “direction”).

**TABLE 2:** Posterior distributions of JAB model parameters, including the paraboloid parameters (mean ± posterior standard deviation) and the environmental variation in log demographic rates (mean ± environmental standard deviation).

Stage	Rate	Parameter	Explanation	Fagus	Others	Prior
J	r	logr	Environmental variation	2.232 ± 0.1561	4.629 ± 0.1366	
		$\epsilon_{r}$	Extremum	2.235 ± 0.1560	4.632 ± 0.1367	4 ± 1
		$\kappa_{r\;x}$	Center (pH)	0.0001603 ± 0.9643	0.1004 ± 0.9937	
		$\kappa_{r\;y}$	Center (soil water)	0.008588 ± 0.9955	0.01348 ± 0.9690	
		$\zeta_{r\;x}$	Spread (pH)	−0.0007368 ± 0.0005630	−0.0007302 ± 0.0005647	
		$\zeta_{r\;y}$	Spread (soil water)	−0.0007352 ± 0.0005440	−0.0007166 ± 0.0005464	
	$c_{J}$	$\log c_{J}$	Environmental variation	−12.23 ± 0.2726	−8.272 ± 0.7036	
		$\epsilon_{c\;J}$	Extremum	−12.79 ± 0.3719	−8.964 ± 0.8795	−11 ± 2
		$\kappa_{c_{J}\;\;x}$	Center (pH)	0.4383 ± 0.6883	0.8527 ± 0.6355	
		$\kappa_{c_{J}\;y}$	Center (soil water)	−0.9838 ± 0.6394	−0.9699 ± 0.6583	
		$\zeta_{c_{J}\;x}$	Spread (pH)	0.1329 ± 0.08853	0.1771 ± 0.1001	
		$\zeta_{c_{J}\;y}$	Spread (soil water)	0.1736 ± 0.09618	0.1731 ± 0.09071	
	s	logs	Environmental variation	−9.367 ± 1.030	−0.3539 ± 0.5606	
		$\epsilon_{s}$	Extremum	−10.25 ± 1.216	−0.5047 ± 0.5613	−6 ± 2
		$\kappa_{s\;x}$	Center (pH)	0.2164 ± 0.7749	−0.1824 ± 0.9156	
		$\kappa_{s\;y}$	Center (soil water)	−0.2101 ± 0.7144	1.039 ± 0.8310	
		$\zeta_{s\;x}$	Spread (pH)	0.2539 ± 0.2006	0.02001 ± 0.01794	
		$\zeta_{s\;y}$	Spread (soil water)	0.3971 ± 0.3607	0.04323 ± 0.02879	
J/A	g	logg	Environmental variation	−5.190 ± 0.1140	−2.304 ± 0.5375	
		$\epsilon_{g}$	Extremum	−4.863 ± 0.1734	−2.227 ± 0.5361	−4 ± 1
		$\kappa_{g\;x}$	Center (pH)	−0.3004 ± 0.7416	−0.2853 ± 0.8740	
		$\kappa_{g\;y}$	Center (soil water)	1.015 ± 0.5867	0.4063 ± 0.9424	
		$\zeta_{g\;x}$	Spread (pH)	−0.06200 ± 0.04883	−0.01870 ± 0.01612	
		$\zeta_{g\;y}$	Spread (soil water)	−0.1155 ± 0.05590	−0.02175 ± 0.01889	
		*d.‐d. term*	At initial state	−5.224 ± 0.1109	−4.895 ± 0.1050	
			At equilibrium	−5.254 ± 0.1090	−6.053 ± 0.2159	
A	$c_{A}$	$\log c_{A}$	Environmental variation	−8.859 ± 1.082	−6.622 ± 0.2118	
		${\epsilon_{c}}_{A}$	Extremum	−9.551 ± 1.328	−6.756 ± 0.2456	−7 ± 2
		$\kappa_{c_{A}\;x}$	Center (pH)	0.1756 ± 0.7395	0.1406 ± 0.8022	
		$\kappa_{c_{A}\;y}$	Center (soil water)	−0.2601 ± 0.8150	0.1530 ± 0.8889	
		$\zeta_{c_{A}\;x}$	Spread (pH)	0.3011 ± 0.2648	0.04833 ± 0.04099	
		$\zeta_{c_{A}\;y}$	Spread (soil water)	0.1924 ± 0.1923	0.03918 ± 0.03615	
A/B	h	logh	Environmental variation	−2.835 ± 0.1221	−2.988 ± 0.07214	
		$\epsilon_{h}$	Extremum	−2.412 ± 0.2077	−2.823 ± 0.08453	−3 ± 1
		$\kappa_{h\;x}$	Center (pH)	0.9856 ± 0.5552	−0.09541 ± 0.8960	
		$\kappa_{h\;y}$	Center (soil water)	0.4131 ± 0.4774	1.089 ± 0.4414	
		$\zeta_{h\;x}$	Spread (pH)	−0.1220 ± 0.06250	−0.01314 ± 0.01197	
		$\zeta_{h\;y}$	Spread (soil water)	−0.1421 ± 0.08160	−0.06805 ± 0.02732	
		*d.‐d. term*	At initial state	−2.841 ± 0.1232	−3.017 ± 0.07450	
			At equilibrium	−2.850 ± 0.1250	−3.062 ± 0.08182	
B	b	logb	Environmental variation	−3.546 ± 1.292	−3.047 ± 0.2228	
		$\epsilon_{b}$	Extremum	−2.947 ± 0.4956	−3.004 ± 0.2172	−3 ± 1
		$\kappa_{b\;x}$	Center (pH)	0.5314 ± 0.7471	0.006300 ± 0.9264	
		$\kappa_{b\;y}$	Center (soil water)	−0.4941 ± 0.9391	0.1035 ± 0.9095	
		$\zeta_{b\;x}$	Spread (pH)	−0.1114 ± 0.1490	−0.009622 ± 0.009471	
		$\zeta_{b\;y}$	Spread (soil water)	−0.1498 ± 0.2490	−0.01575 ± 0.01439	
		*d.‐d. term*	At initial state	−3.576 ± 1.281	−3.078 ± 0.2171	
			At equilibrium	−3.614 ± 1.271	−3.126 ± 0.2115	
B	$c_{B}$	$\log c_{B}$	Environmental variation	−6.754 ± 0.6812	−6.543 ± 0.1965	
		${\epsilon_{c}}_{B}$	Extremum	−6.916 ± 0.7710	−6.600 ± 0.2057	−7 ± 2
		$\kappa_{c_{B}\;x}$	Center (pH)	0.1291 ± 0.8907	0.4474 ± 0.8884	
		$\kappa_{c_{B}\;y}$	Center (soil water)	−0.3808 ± 0.8419	0.4679 ± 0.8594	
		$\zeta_{c_{B}\;x}$	Spread (pH)	0.04258 ± 0.04792	0.01304 ± 0.01124	
		$\zeta_{c_{B}\;y}$	Spread (soil water)	0.05732 ± 0.07456	0.01776 ± 0.01516	
(J)	$L_{p}$	$\log L_{p}$	Environmental distribution	2.428 ± 0.1205	7.656 ± 0.1531	
		logl	Constant across environmental	1.691 ± 0.1205	5.095 ± 0.1531	5 ± 2

*Note:* Demographic rates are generated by the corresponding paraboloid parameters ϵ, κ, ζ with the respective environmental axes x (pH) and y (soil water level). In addition to the growth rates logg, logh and logb the corresponding density‐dependent (*d.‐d*.) terms across all subpopulations are given at initial and equilibrium state, that is, $\log\left(g⊘\left[1+c_{J}\;\mathrm{sum}\left(J_{t}\right)+s\;\mathrm{sum}\left({\mathrm{BA}}_{t}\right)\right]\right)$, $\log\left(h⊘\left[1+c_{A}\;\mathrm{sum}\left({\mathrm{BA}}_{t}\right)\right]\right)$, and $\log\left(b⊘\left[1+c_{B}\;\mathrm{sum}\left({\mathrm{BA}}_{t}\right)\right]\right)$.

For a detailed explanation of the JAB model, see the description in Supporting Information A.1 and Figure S1, reproduced from Heiland et al. (2023). For a discussion of its utility and limitations, see Section 4.3.

### 2.2 Short Time Series From National Forest Inventory Data

We fitted the JAB model with short time‐series of repeated tree counts and circumference measurements from the German national forest inventory (NFI; main years 1987, 2002, 2012; Table S1, Figure S2).

The plots of the German NFI are arranged on a regular quadratic grid with clusters that are 4 km apart, each consisting of four plots that are 150 m apart (Tomppo et al. 2010). In some federal states, the grid density may be increased to 2.83 km or 2 km between clusters. For fitting the JAB model, one plot per cluster was selected randomly to represent a subpopulation.

Trees in stage J, that is, saplings between height 20 cm and dbh 7 cm were counted on circular fixed area plots with radii dependent on size class and survey (Table S2). Trees above 10 cm diameter at breast height (dbh), corresponding to stages A and B, were sampled using the angle count method (Riedel et al. 2017; Kangas and Maltamo 2006) (for a detailed description see Section S2.2). Instead of a fixed area for all tree sizes, the selection with an angle leads to a sampling area that varies with dbh. To account for this, we introduced dbh‐dependent offsets (see Section 2.4.3; Section SA.5).

The theory tested here focuses on the performance of the competitive species *Fagus sylvatica* against the average of other species within a specific environment. To simplify computation, instead of estimating demographic rates for each species, we modeled all competitors of *Fagus sylvatica* as a single group. This approach results in two groups, called in the modeling context: the species *F. sylvatica* and all *other* species (for discussion of the consequences of grouping *others*, see Section 4.3.2).

To ensure the applicability of the NFI data to Ellenberg's original predictions, we selected 795 plots using stratified random sampling based on the availability and balance of environmental variables. Plots were restricted to elevations between 100 m and 600 m, corresponding to the submontane range relevant to Ellenberg (1963), and included only those with complete survey timelines (1987, 2002, 2012) that remained undisturbed after the initial survey. For details, see Section SA.3.

### 2.3 Environmental Variables

We studied predominance of *Fagus* along the environmental gradients soil pH and soil water level, matching the original gradients in Ellenberg (1963). These gradients are viewed as proxies for multiple underlying environmental variables driving species turnover (Ellenberg 1963). Soil pH, for example, is a general proxy for plant nutritional conditions because it is tightly correlated with factors such as base saturation and C/N ratios (Härdtle et al. 2004). Therefore, we did not explore any additional environmental dimensions. For fitting the JAB model, we scaled and centered both soil variables. To visualize plot‐level data in two‐dimensional environmental space, we added a consistent uniform jitter [–0.1, 0.1] around the unscaled variables. Further, to put the environmental range of the German NFI into perspective, we compared it with *Fagus sylvatica* 's European range of distribution (Figure S3).

For soil pH, we used the top soil pH in ${\text{CaCl}}_{2}$, provided as a spatial raster at 500 m resolution by the European Soil Data Centre (Ballabio et al. 2019). Values of the spatial raster were extracted based on the coordinates of the forest plot locations.

Soil water data was derived from hydromorphic soil water and groundwater categories from the National Forest Soil Survey (Benning et al. 2019) at locations of the German NFI to match the soil water gradient of Ellenberg's prediction. The categories were ranked according to the levels in Ellenberg (1963) based on expert knowledge and the category descriptions in Benning et al. (2016). To achieve a pseudo‐ratio scale, the ordered categories were assigned rational numbers so that the levels between (−6.0), (0.0), and (8.0) were uniformly spaced (see Table S3 for ranking details). The resulting values were averaged per plot, weighted by the respective soil layer thickness.

### 2.4 Fitting the JAB Model With Environmentally‐Responsive Demographic Rates

#### 2.4.1 Model Subpopulation Structure

In the JAB model, each forest plot is represented as a subpopulation that changes over time. Each subpopulation has random initial states of the three stages (J, A, and B), which are fitted as parameters to the first observed states of each plot, and then simulated forward (for prior distributions see Section SA.6). The forward‐simulated annual model states are matched to the observed data, with the discrete time t after the initial state t=1 in the model to the *t*th year after the first data survey (e.g., 1987). The subpopulation‐specific demographic rates (Table 1) are generated dependent on the respective environmental variables (Section 2.4.2).

#### 2.4.2 Environmental Response of Demographic Rates

Environmental variability of the JAB model rates (r, $c_{J}$, s, g, $c_{A}$, h, b, and $c_{B}$) is achieved by fitting bell‐shaped paraboloids dependent on the two environmental variables x (soil pH) and y (soil water level). The parabolic response of a demographic rate (f) at a subpopulation (p) is expressed as the sum of two quadratic response curves,

(1) $$f_{p}=\exp\zeta_{x}{\left(x_{p}-\kappa_{x}\right)}^{2}+\zeta_{y}{\left(y_{p}-\kappa_{y}\right)}^{2}+\epsilon ,$$

where ϵ corresponds to the optimum of a demographic rate (maximum of the growth‐related rates r, g, h, and b, and minimum of the competition effects $c_{J}$, s, $c_{A}$, and $c_{B}$). The positions of these optima along the two environmental axes x and y are given by κ and the two respective spreads over the axes are given by ζ. The exponentiation ensures that all JAB model rates are strictly positive.

This vertex parameterization, with five paraboloid parameters per demographic rate, makes it straightforward to formulate prior distributions. We specified equivalent priors for both populations that had the highest probability at a flat response to the environment (for details see Section SA.7).

In contrast to the other demographic rates, the seedling input rate l varies not with the environmental variables, but with a spatial smooth of the regional conspecific basal area, a measure for long‐distance and long‐term dispersal (details in Section SA.4). The regional basal area $B_{p}$ informs the subpopulation‐specific values, $L_{p}=\exp\left(\log l\right)B_{p}$ iterated over the subpopulations or NFI plots p.

To characterize the variation in demographic rates along environmental gradients, we took the means of the posterior log rates per subpopulation and summarized this *environmental variation* by calculating means and standard deviations (Table 2).

#### 2.4.3 Likelihood Function

All tree abundance data from the German NFI arise from a count process, including basal area per hectare, which is obtained via angle count sampling. We therefore fitted the JAB model states, including the unknown initial state, with Negative Binomial likelihood functions with variable offsets and observation errors.

Different offsets were used to integrate two kinds of data, both from count processes (see Section SA.5 for details): (1) Count data in the size classes J and A originate from a count process on varying areas per tree size, so that the area‐standardized model state (counts per hectare) is related to the observations with an offset o, which is the average area of one sampling plot. (2) Basal area data in size class B also originate from a count process of individual trees with varying sampling probabilities and areas dependent on their dbh (angle count sampling; Section SA.5), but in addition, each counted tree also carries an individual basal area record. Hence, to relate the model state of B (basal area per hectare) to observed counts in size class B, we use a special offset that includes both the average sampling area and a factor $\frac{c_{p}}{{\mathrm{ba}}_{p}}$, which expresses how many counts $c_{p}$ are added per unit of basal area ${\mathrm{ba}}_{p}$ on average per plot. The offsets o transform the area‐standardized and strictly positive model states (JAB) to the counts C in the data, so that for each subpopulation p, year t (or survey r), population j, and stage s, the likelihood function is expressed by:

(2) $$C_{\text{ptjs}}∼\text{NegativeBinomial}\left({\mathrm{JAB}}_{\mathrm{tjs}}\;o_{\text{ptjs}},\phi_{\mathrm{rjs}}\right).$$

We accounted for differences in sampling protocols and areas by allowing observation errors, that is, precision parameter ϕ, to vary between stages, populations, and surveys (Table S6). For the initial state, which was initialized based on the data of the first survey, a different level for ϕ was assumed (Table S6). Additionally, changes in sampling areas for size class J between surveys required different values of ϕ for all three surveys in this class. As a result, there were six levels of ϕ in stage J, four levels in stage A, and four levels in stage B. A half‐normal prior for the inverse of ϕ was used to improve convergence: $\frac{1}{\phi }∼H\left(1\right)$.

#### 2.4.4 Model Fitting

The model was fitted with stan (version 2.31.0; Stan Development Team 2022) using the standard Hamiltonian Monte Carlo algorithm (for details see Section SA.8). Model complexity was determined through staged model‐building on the same dataset (Table S1): starting from an initial model with simple demographic rates, we incrementally added a paraboloid environmental response to each demographic rate until sampling time reached approximately 8 h.

### 2.5 Posterior Simulations From the Fitted JAB Model

Using the posterior probability distributions of the demographic rates, we simulated competitive equilibria and the effect of environmentally‐responsive demography on relative abundance.

#### 2.5.1 Competitive Equilibria

**FIGURE 2:**
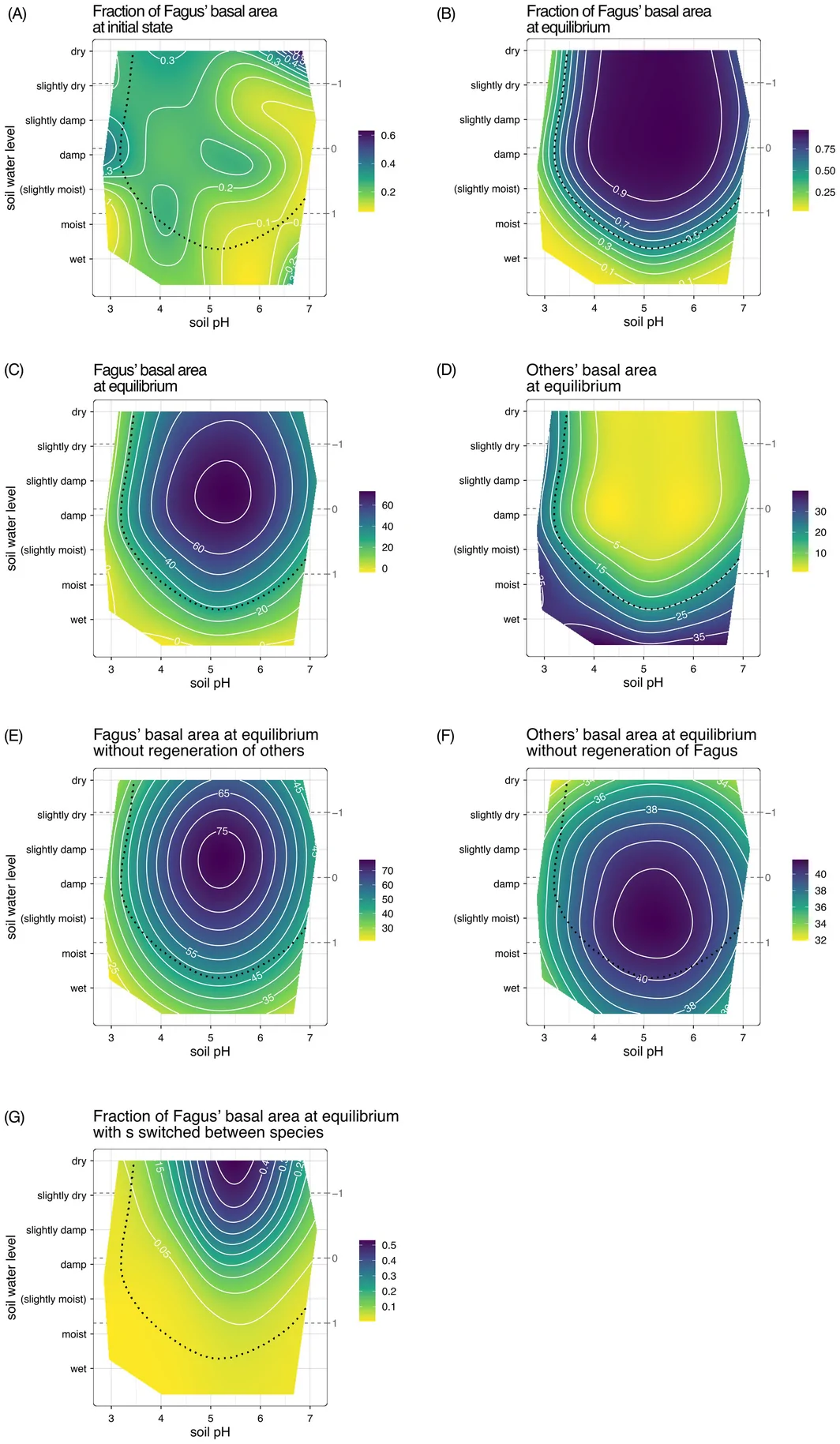
Basal areas in environmental space at initial state (A), at the competitive equilibrium (B–D). Additionally, counterfactual equilibria are depicted: Given that the respective other population does not reproduce (by setting l, r, and b to 0; E–F), and (G) given that the shading response (s) is switched between *Fagus* and *others*. The contours show predictions from smoothing splines fitted to the posterior distributions (as described in Section 2.5.1).

To test the prediction, that *Fagus* will be the predominant population at mesic environmental conditions, we simulated the JAB model timelines forward from the initial state, until equilibrium states were reached. We considered the equilibrium to be reached when the greatest population‐specific relative change in basal area (BA) in the last time step did not exceed 1: $\max|\left({\mathrm{BA}}_{t}-{\mathrm{BA}}_{t-1}\right)⊘{\mathrm{BA}}_{t}|\leq 0.001$. This equilibrium criterion is equivalent to the established procedure for numerical solution of fixed‐points of a function as iteration rule (Burden and Faires 1993). The model was simulated forward at least 250 years to ensure that temporary extrema at the beginning of the trajectory were not mistaken as equilibria (Heiland et al. 2023), and for a maximum of 6000 years. Additionally, we generated counterfactual equilibria where the regenerative rates (l, r, b) of the respective other population were set to 0, to show the potential abundance of a population in the absence of the other population (Figure 2). Further, to demonstrate the role of the shading parameter s, which had been shown to be the most important for predominance at equilibrium overall (Heiland et al. 2023), we simulated equilibria with s switched between *Fagus* and *others* (Figure 2G).

Equilibrium abundances were visualized by fitting and predicting two‐dimensional tensor smoothing splines along the two environmental axes using the gam function of the mgcv package (version 1.8; Wood 2021). The model was fitted to the posterior distribution with a Gaussian and binomial response for population‐specific basal area and its fraction of total basal area, respectively. Predictions from the model were visualized only within the convex hull that included the observed plots, to avoid extrapolation. The line indicating the range of *Fagus* predominance was visualized by predicting the isocline of 50% relative basal area at equilibria (Figures 1, 2B).

#### 2.5.2 Effect of Environmentally‐Responsive Demography on Relative Abundance at Equilibrium

To quantify the effect of environmentally‐responsive demography on the competitive equilibria, we calculated the differences in *Fagus*' relative basal area at the equilibria with and without environmental variation in the different demographic rates. To simulate counterfactual equilibria without environmental variation in a certain rate, we used the mean rate across subpopulations for simulating all subpopulations. To evaluate the effects that an environmentally‐responsive rate has on *Fagus*' relative abundance, we computed the differences of the basal area fraction that *Fagus* has at equilibria with a flat mean rate versus an environmentally‐responsive rate. Negative differences indicate that an environmentally‐responsive demographic rate decreases *Fagus*' basal area at equilibrium. In Figure 4, we summarize the *effect of environmentally‐responsive demography* for each demographic rate, represented by the mean of the absolute differences.

We further visualized the effect of environmentally‐responsive rates in environmental space using Gaussian smoothing splines, as outlined in Section 2.5.1 (Figure 5 and Figures S8 and S10). To identify credible effects, we determined the fraction of the posterior per subpopulation that was greater or smaller than 0. If more than 90% of the posterior met either condition, we determined the effect of environmentally‐responsive demography of a subpopulation as credibly positive or negative, respectively.

## 3 Results

### 3.1 Equilibria in Environmental Space

At the equilibria simulated with inferred environmentally‐responsive demographic rates, *Fagus* was most abundant and predominated (basal area fraction > 50%) in the central region of the environmental space, that is, at mesic environmental conditions (Figure 1, Figure 2). At the initial state, however, *Fagus* had only 14% of the basal area on average (Table S7), with the highest fractions of *Fagus* instead found at marginal regions (Figure 2A). Equilibrium basal area of *Fagus* decreased towards the margins, especially at the wet and at the acidic margin, resulting in the population of *other* species having higher relative basal area. However, at the dry margin, the relative equilibrium basal area of *Fagus* showed minimal decline.

In counterfactual equilibria where regeneration of *others* was set to zero, *Fagus* remained most abundant in the central region of the environmental space. However, in the absence of *Fagus* regeneration, *others* no longer exhibited the highest abundance at the margins but instead showed similarly high abundance in the central region previously occupied by *Fagus*. Simulating equilibria while switching the shading response (s) between *Fagus* and *others*, revealed that without the high shade‐tolerance of its saplings *Fagus* would never become the predominant population (Figure 2G).

### 3.2 Environmental Variation in Demographic Rates

**FIGURE 3:**
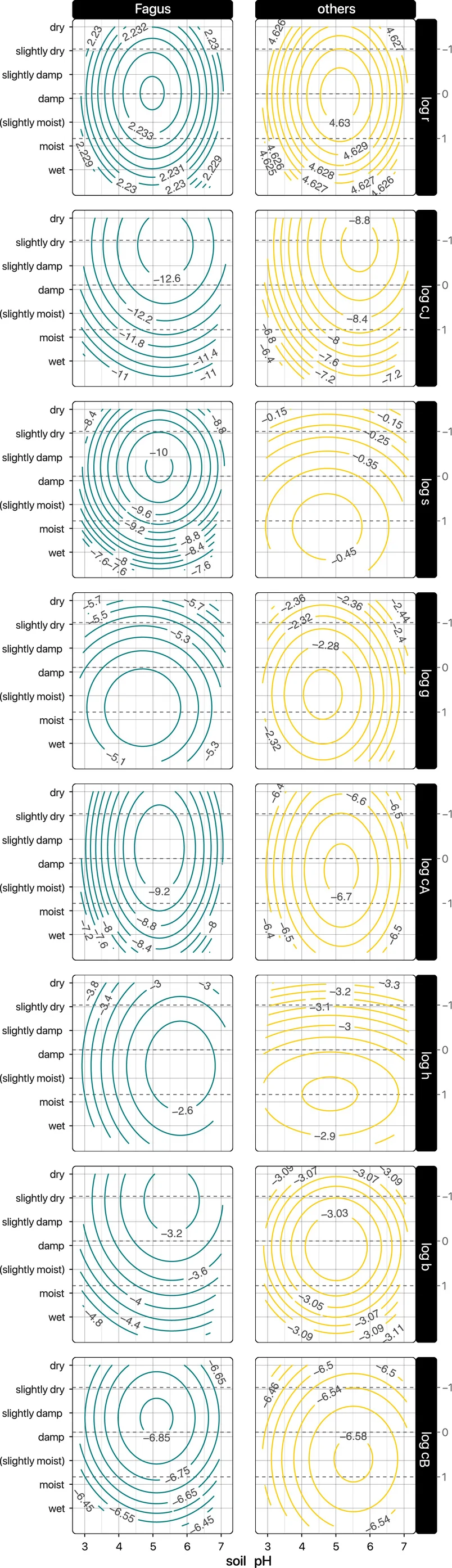
JAB model demographic rates in environmental space. The contours show the mean predicted values from the posterior distribution of polynomials (Section 2.4.2).

In addition to the differences in mean demographic rates between *Fagus* and *others* (Section SB.1), *Fagus* showed much greater environmental variation in its demographic rates compared to *others*. Especially, the demographic rates at the overstory stages A and B had a greater environmental standard deviation on the log scale (environmental variation in Table 2, resulting paraboloids in Figure 3). At the sapling stage J, however, the environmental variation was more similar between populations.

The most significant environmental variation overall was observed in the demographic rate logb of *Fagus*, which includes both basal area growth and density‐independent mortality in overstory stage B, ranging from an optimum of −3.5 (Table 2) to values below −4.8 (Figure 3). The competition response of *Fagus* in stage B ($\log c_{B}$) also varied from a minimum of −6.75 to values below −6.45. In the smaller size stages, the limiting rates $\log c_{A}$ (stage A), and logs, $\log c_{J}$ (stage J) also showed higher variation than the population growth rates, with standard deviations up to 1 on the log scale.

For both populations and most demographic rates, the optimum was positioned near the center of both environmental gradients (Figure 3). Along pH, the optimum for all demographic rates was located close to the center, around moderately acidic values of pH 4.5–6, with a slight tendency towards less acidic conditions indicated by positive $\kappa_{x}$ (Table 2). In contrast, along the soil water gradient, there was more variation in the position of the extrema across populations, as reflected by larger absolute values of $\kappa_{y}$.

The density‐dependent growth terms (Section 2.1) exhibited variations in environmental space similar to the corresponding growth rates (h and b; Figure S7). The density‐dependent transition term for saplings $\frac{g}{1+s\mathrm{sum}\left(\mathrm{BA}\right)+c_{J}\mathrm{sum}\left(J\right)}$, however, had a minimum at the center under equilibrium conditions, reflecting the pattern of the two limiting rates s and $c_{J}$ together with the equilibrium abundance, instead of g (Figure S7).

### 3.3 Effect of Environmentally‐Responsive Demography on *Fagus*' Relative Abundance

**FIGURE 4:**
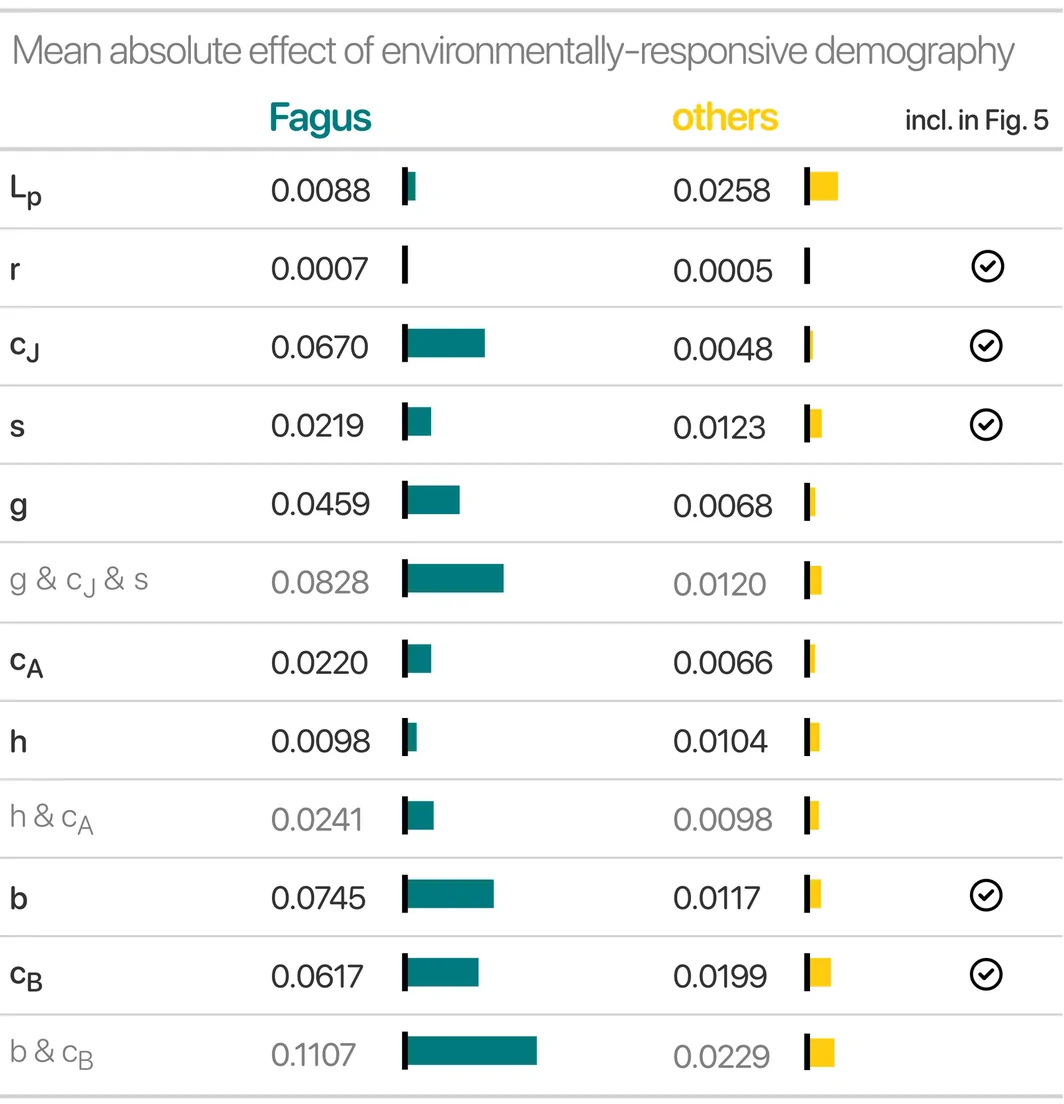
Magnitude of the environmental variation effect per demographic rate (black) and the combined effects of rates in density‐dependent terms (gray). The magnitude is indicated by the mean of the absolute effects.

The environmental response of *Fagus*' demographic rates had a much larger effect on relative abundance compared to *others*, that is, greater absolute mean difference in *Fagus*' relative basal area at equilibria with environmentally‐responsive demographic rates compared to equilibria with flat mean rates (Figure 4). The strongest effects on relative abundance were observed in *Fagus*' overstory stage B, specifically in net basal area increment b and the limiting rate $c_{B}$. The combined environmental response of these two rates also had the largest overall effect on relative abundance. The second‐strongest effect was observed at the sapling stage J, in the rate $c_{J}$. The combination of environmental responses of g and of the counteracting rates $c_{J}$ and s also had a considerable effect on relative basal area. In contrast, at the intermediate size stage A, the environmental responses of growth (h) and competition ($c_{A}$) were not as influential. Interestingly, for *Fagus*, both the seedling recruitment (r) and seedling input rate ($L_{p}$) had minimal influence.

In *others*, environmental responses had considerably smaller effects on abundance compared to *Fagus* but in the overstory the relative ranking of the rates was similar. Specifically, the response of the rate $c_{B}$ had the most significant effect on relative abundance, followed by b. Additionally, in *others*, the environmental variation in seedling input $L_{p}$ and the shading parameter s had the greatest effect on relative abundance at equilibrium. Interestingly, across both populations the environmental response of seedling recruitment r had the weakest effect.

**FIGURE 5:**
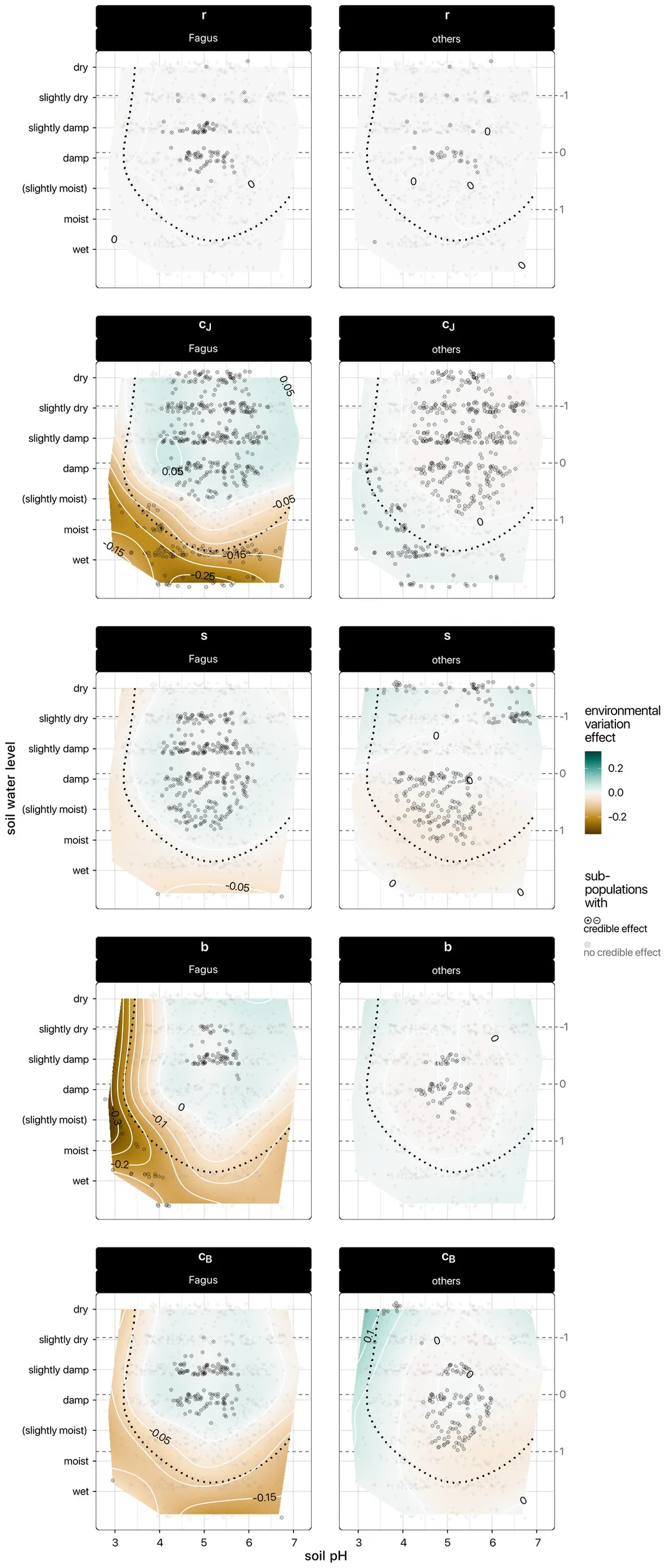
The effect that the environmental response of demographic rates (r, $c_{J}$, s b, and $c_{B}$) has on *Fagus*' relative abundance at equilibrium. The effect of a rate is quantified as the difference between the relative abundances of *Fagus* with and without environmental response of that rate (Section 2.5.2). Dark point characters indicate credible effects. Effects are assumed as credible if the difference is either > or < 0 with a probability of > 90%. The dashed line indicates the range of *Fagus*' predominance.

The environmental responses of b, $c_{J}$, and $c_{B}$ in *Fagus* explain its decreasing basal area at the margins (Section 3.2). The environmental response of these rates had a positive effect on *Fagus*' relative basal area in a central region within its range of predominance, but a negative effects in marginal regions (credibly positive or negative effects—90% of the posterior differences > 0 or < 0 are only present for subpopulations at the core or the very edge of the range, see Figure 5, Section 2.5.2). In contrast, the environmental response of the sapling transition rate g in *Fagus* (Figure S8), did not result in lower relative basal area under moist conditions but only towards dry conditions. In addition, the response of g exhibited a strong positive effect under moist and more acidic conditions.

For both *Fagus* and *others*, joint effects of multiple environmentally‐responsive rates had an amplified pattern compared to the individual rates alone, for the combination of sapling rates g, $c_{J}$, and s, combined rates of the intermediate stage h and $c_{A}$, as well as the combined rates of the final stage b and $c_{B}$. Specifically, the combined effects had a greater positive and negative magnitude, as well as higher credibility compared to their individual effects (Figure S9).

For the environmental patterns of the response effects of all rates see (Figure S8).

## 4 Discussion

In this study, we aimed to test Ellenberg's classical ecological theory on species composition in Central European forests (Ellenberg 1963) by Bayesian inverse calibration of a size‐structured forest population model, including two populations, that is, *Fagus sylvatica* and all *other* species. Our simulations largely support the prediction that *Fagus* predominates in mesic environmental conditions while *other* species become more prevalent at the extremes of soil pH and water gradients (Section 4.1). Moreover, we substantiate the theory with demographic analyses (Section 4.2): In a previous study, Heiland et al. (2023) showed that the shade‐tolerance of its saplings explained *Fagus*' predominance in its core range. Expanding on this approach, here we introduce a model with environmentally‐responsive demographic rates to explain the environmental variation in *Fagus*' relative abundance and why *other* species prevail towards more stressful environmental conditions. Specifically, we confirm that the high shade‐tolerance of *Fagus*' saplings is key for its dominance in mesic environments but that the environmental variation in overstory demography is greater than that in saplings. The simulations show that the environmental response of adults' rather than saplings' demographic rates explains the decrease in *Fagus* relative abundance in extreme environments.

### 4.1 Are Simulated Equilibrium Abundances Along Environmental Gradients Consistent With Theoretical Predictions?

#### 4.1.1 *Fagus* Predominates at the Center of Environmental Space

Our findings provide evidence for Ellenberg's theory of *Fagus* predominance. At the simulated competitive equilibrium, *Fagus* dominates under mesic conditions in a central to dry region of the environmental space while declining towards the margins (Section 3.1; Figure S3). Our simulations deviate only slightly from the original predictions of Ellenberg (1963) who postulated a central range of predominance for *Fagus* with species turnover at the dry margins (Figure 1). Our results show a minimal decline of *Fagus*' abundance under dry conditions, likely due to three data limitations at the dry edge: First, in contrast to Ellenberg's formulation, the scale available to this study only has the level and does not differentiate (Benning et al. 2019; Table S3; Ellenberg 1963). The lacking differentiation may also have been caused by the much higher number of sampled forest plots at the dry compared to the wet margin (Table S4). Second, the sampled data range covers a relatively moist portion of *Fagus*' range, as evident from the distribution of climatic water balance of the German NFI plots compared to the broader range in the European Atlas of Forest Tree Species (means and standard deviations: 215.8 ± 193.5 vs. 158.3 ± 343.9 mm a^−1^; Figure S3; de Rigo et al. 2016). Third, there may be a selection bias in the cultural landscape where extremely dry areas that could be forested (typically with predominance by *Quercus* spp.), have consistently been turned into grasslands throughout Germany (Poschlod 2015). Consequently, such areas may be excluded from the forest sample, although they have influenced Ellenberg's theory about natural forests.

In general, both *Fagus* and *others* have a broader distribution along the soil water gradient (Figure 2), which is at least partially caused by the broader distribution of rate optima along soil water compared to pH (Table 2). The joint effect of broadly distributed optima of the demographic rates results in a more gradual decline towards the margins and a broader abundance along this gradient (Sharma et al. 2022).

The simulated total equilibrium basal area of around 57 m^2^ ha^−1^ (Table S7) is only slightly higher than expected from observations. Stillhard et al. (2022) report total basal areas of ∼36 m^2^ ha^−1^ from pristine beech‐dominated forests in Uholka‐Shyrokyi Luh, Ukraine, while Moreno et al. (2018) project an upper limit of European forests around 50 m^2^ ha^−1^ (see also Heiland et al. 2023).

Nevertheless, considering that our model does not account for disturbance events like fires, storms, and pathogen damage, the extrapolated equilibria fall within a realistic range and can serve as external validation of the model (e.g., Cailleret et al. 2020).

#### 4.1.2 In *Fagus*' Absence, *Others* Would Also Be Most Abundant at the Center of Environmental Space

In a counterfactual simulation where *Fagus* abundance was minimized by preventing its regeneration, *others* showed maximum abundance in the central region otherwise occupied by *Fagus* (Figure 2F). This demonstrates that *others* also have their physiological optimum at the center of the environmental space but are outcompeted by *Fagus*. This mechanism, whereby most species have their physiological optimum under intermediate conditions but species interactions drive the species turnover along environmental gradients (ecological optimum), is a central principle of Ellenberg's original theory (Ellenberg 1952; Ellenberg 1963; see also Hutchinson 1957; Keddy 2001). The theory also aligns with the stress‐gradient hypothesis (Bertness and Callaway 1994; Käber et al. 2023), which proposes that as stress decreases in favorable conditions, competition intensifies, as supported by empirical findings on *Fagus* by Meier et al. (2011). Consequently, it can be concluded that *Fagus* predominates because its demography is disproportionally more beneficial at the center so that it outcompetes *others* (see also Jacobs et al. 2022).

### 4.2 How Does the Environmental Response of Demographic Rates Control Relative Abundance?

#### 4.2.1 Environmental Variation in *Fagus*' Relative Abundance Is Driven by the Environmental Response of *Fagus*' Demographic Rates Rather Than *Others*

Demographic rates of *Fagus* vary more strongly along the environmental gradients compared to *others* (Section 3.2). As a consequence, *Fagus*' relative abundance and therefore predominance in mesic environmental conditions is primarily driven by the environmental response of *Fagus*' demographic rates rather than the response of the grouped *other* species (Figure 4, Section 3.3). That *Fagus*' own demographic rates drive its variation in abundance aligns with expectations for a dominant species.

Although the environmental variation in the log demographic rates (Table 2) already hints at the impact on the environmental variation in *Fagus*' relative abundance, the actual effect that the demographic rates have on equilibrium abundance is also dependent on the interaction with other processes in the JAB model. To account for these interactions, we quantified environmental effects by comparing equilibria with the given environmentally‐responsive demographic rates to equilibria with flat mean demographic rates (Section 2.5.2, Figure 5). The three most important effects of environmentally‐dependent demography on relative abundance relate to *Fagus*': b, $c_{J}$, and $c_{B}$ (Figures 4 and 5). Among these, environmental response of the net basal area increment b had an exceptionally negative effect on *Fagus*' relative abundance at the acidic edge. This finding aligns with a study by Aertsen et al. (2012), which reports a sharp decrease in *Fagus*' adult growth when the soil pH drops below 3.6, likely due to aluminum toxicity. In turn, the effect of b, which includes both basal area growth and density‐independent mortality, does not lead to a significant decrease of abundance at the dry edge. This may be attributed to *Fagus*' moderate drought tolerance (Rötzer et al. 2017; Leuschner 2020), particularly compared to the most common *other* species, *Picea abies* (L.) H. Karst. (Niinemets and Valladares 2006; Rötzer et al. 2017; Pretzsch et al. 2020; see Table S5 for species composition). Various studies have, however, demonstrated increased mortality under severe drought (Ciais et al. 2005; Geßler et al. 2006; Leuschner 2020), especially at the southern margin of *Fagus*' distribution (Jump et al. 2006; Archambeau et al. 2020, here density‐independent mortality). The insignificant effects of environmentally‐responsive demography observed at the dry margin could also be due to the limitations of the soil water scale discussed in Section 4.1. Towards wet conditions, the negative effect of *Fagus*' environmentally‐responsive b becomes more pronounced, which aligns with reports about *Fagus* growth sensitivity to waterlogging (Dreyer 1994; Geßler et al. 2006; Ferner et al. 2012; Durrant et al. 2016; though see Scharnweber et al. 2013).

The effect of *Fagus*' environmentally‐responsive $c_{B}$ on relative abundance, which represents the adult competition response including mortality and growth reduction, follows a similar pattern as b with severe negative effects in wetter conditions (see Fichtner et al. 2012). However, $c_{B}$ exhibits less severe negative effects at the acidic margin (Figure 5). The pattern at the dry margin aligns with findings by Jacobs et al. (2022), who also reported no increased competition effect on *Fagus* growth under drier conditions.

Saplings' response to competition by all saplings ($c_{J}$) is an important process capturing mortality in the JAB model, besides the shading response s. $c_{J}$ is solely dependent on the density within the sapling stage J itself. This may lead to the environmental response of the rate $c_{J}$ also capturing environmental variation due to density‐independent mortality, since the JAB model disregards density‐independent mortality at stage J (Section 4.3). Consequently, here we discuss $c_{J}$ as a proxy for both density‐dependent and density‐independent mortality—also given that the literature on environmental variation in *Fagus* sapling mortality does not differentiate between the two. In our study, the environmental response of *Fagus*' $c_{J}$ showed positive effects on its relative abundance under neutral and dry conditions, but highly negative effects in acidic and wet conditions. In line with this, a study by Wilkens and Wagner (2021) emphasizes the role of soil nutrient availability for increased survival of small beech saplings, whereas very acidic conditions indicate low soil nutrients (Härdtle et al. 2004, but see Leuschner and Ellenberg 2017). Also in agreement with our results at the upper end of the pH gradient, Härdtle et al. (2004) found no major negative effects on *Fagus* saplings under a liming treatment (Ljungström et al. 1990). Regarding drought tolerance, while Fotelli et al. (2003) found that beech seedlings are more tolerant to dry conditions compared to other co‐occurring woody species (see Madsen and Larsen 1997), while Tomasella et al. (2019) highlight their susceptibility to drought. However, excess of soil ground water, also decreases the probability of *Fagus* sapling occurrence (Axer et al. 2021), likely due to disadvantageous carbon allocation under waterlogging compared to other species (Dreyer 1994; Schmull and Thomas 2000).

#### 4.2.2 Environmental Response of Overstory Demographic Rates Is More Important Than the Environmental Response at Earlier Stages

In less favorable environments the response of demographic rates in adult trees (b and $c_{b}$ in stage B) more strongly decreased *Fagus* relative abundance than earlier stages. Specifically, the effect of combined environmental responses at the overstory stage (b combined with $c_{B}$) was greater than the combined effect of rates at the sapling stage (g with s and $c_{J}$). This finding may initially seem surprising, as a previous study has shown that the lower response of *Fagus* saplings to shading (s) is the primary factor driving the predominance of *Fagus* at the competitive equilibrium (Heiland et al. 2023; see also Petritan et al. 2007; Leuschner and Ellenberg 2017; Petrovska, Bugmann, et al. 2021). However, this apparent contradiction can be resolved. While variation in shade‐tolerance is not the main driver of the species' abundance response in environmental space, the mechanism of shade tolerance remains crucial for *Fagus* predominance in the first place. This is supported by simulated equilibria where the shading response (s) was switched between *Fagus* and *others* (Figure 2G). The smaller effect observed in the environmental response of seedling and sapling rates aligns with the theory that the occurrence of a particular life stage is constrained by the presence of earlier life stages due to or (Heiland et al. 2022; Young et al. 2005; Ramachandran et al. 2023). Therefore, earlier tree life stages often have broader environmental distributions, as shown by Bertrand et al. (2011), Zhu et al. (2014), Máliš et al. (2016), Copenhaver‐Parry et al. (2020).

### 4.3 JAB Modeling Approach

#### 4.3.1 Advantages of the Process‐Explicit Approach

The JAB model is a process‐explicit demographic abundance model (Briscoe et al. 2019), designed to explain species abundance along environmental gradients by modeling underlying demographic processes across life stages (Heiland et al. 2023). This approach goes beyond simple correlations by capturing mechanistic links between environment, demography, and population dynamics.

Process‐explicit models help identify the relative importance of demographic processes and species interactions in different environments (Schurr et al. 2012; Thuiller et al. 2013; Malchow et al. 2022). For trees, early life stages are critical filters for community assembly (Grubb 1977; Young et al. 2005; Lines et al. 2019), and saplings often respond differently to the environment than adults (Bertrand et al. 2011; Heiland et al. 2022). Therefore, explicit modeling of recruitment and early‐stage performance is essential to capture demographic variation across gradients (Price et al. 2001; Kunstler et al. 2009; Hanbury‐Brown et al. 2022; König et al. 2022).

While process‐explicit models were recognized for their potential to predict distributions under climate change (Buckley et al. 2010; Pagel and Schurr 2012; Zurell et al. 2012; Thuiller et al. 2013; Merow et al. 2014), they were not widely adopted due to difficulties in parameter estimation and limited gains in predictive power over correlative species distribution models (SDMs) (Morin and Thuiller 2009; Zurell et al. 2016; Briscoe et al. 2019).

The JAB model overcomes key limitations of typical process‐based forest models by enabling inverse calibration to short time series of population states, without requiring detailed demographic data on mortality, growth, and regeneration. This approach combines the accessibility of correlative models with the explanatory power of process‐explicit models (Dormann et al. 2012).

#### 4.3.2 Limitations

The parsimonious structure of the JAB model enables easy calibration but also entails limitations.

First, the model includes density‐independent mortality only in the net increment b of stage B, while stages J and A experience only density‐dependent mortality (see Heiland et al. 2023). This simplification was necessary because, without additional demographic information, density‐independent mortality is difficult to distinguish from seedling input. Ignoring density‐independent mortality might lead to overestimating competition effects by erroneously absorbing variation due to density‐independent mortality, or underestimating growth rates in stages J and A compared to a system with density‐independent mortality (see also Section 4.2.1). Future work could improve the model by additionally including density‐independent mortality, using direct estimation of informative priors on mortality and growth rates (Heiland et al. 2023; Hartig et al. 2012).

Second, fitting the model solely to time series of abundance states introduces potential equifinality, where different parameter configurations can produce similar model outputs that fit the data (Hartig et al. 2014). In the JAB model this was evident through correlations in the posterior estimates of specific rates, such as the optima of overstory rates $\epsilon_{b}$ and $\epsilon_{\mathrm{cB}}$ (Figures S4–S6). These equifinalities would be a problem for interpretation, if the correlated rates would cancel each other out. To confirm that this is not the case, we check all demographic rates that jointly control one stage. This involves comparing the estimates with the full density‐dependent growth terms, for example, $g⊘\left[1+c_{J}\;\mathrm{sum}\left(J_{t}\right)+s\;\mathrm{sum}\left({\mathrm{BA}}_{t}\right)\right]$ (Table 2) and jointly averaging parameters to compute the effect of their joint environmental response (Section 4.2, Figure 4, Figure S9).

Third, the paraboloid response (Section 2.4.2) lets each demographic rate vary at most unimodally along each environmental axis, and, because the two quadratic terms are additive, independently along the two. The elliptic contours of a rate's response therefore always align with the pH and soil water axes, so that a rate cannot, for example, tolerate acidic conditions better on moist than on dry soils; such a correlated response would correspond to a rotated ellipse. Capturing it would require an interaction term, at the cost of one further parameter per demographic rate and population with increased sampling difficulty. We refrained from adding this complexity because our hypotheses concern the overall magnitude of each rate's environmental response rather than the orientation of its contours (Section 2.5.2), and because a joint dependence on pH and soil water emerges in any case at the level of relative equilibrium abundance, which results from the interplay of the eight demographic rates and the two populations. Adding correlated responses would nevertheless be the natural next refinement of our approach towards more realism.

Fourth, the model simplifies interspecific competition by aggregating all non‐Fagus species into a single group of species. This way, *others* can be interpreted as a background population with average demography at a specific environment. While there is one average estimate for *others* per demographic rate, this estimate is allowed to vary along environmental gradients. Consequently, the demographic variation in *others* reflects both species' variable demographic performance and the turnover of demographically different species along those gradients. This species turnover is supported by the more broadly distributed optima of different demographic rates observed in *others* compared to *Fagus* (Section 4.1.1). Because demographic rates for *others* are environmentally averaged, they cannot be linked to individual species. We therefore focus our conclusions on the relative abundance of *Fagus* compared to the average background population under the specific environment. Since competition with *Fagus* may shift species composition towards more competitive species, assuming a static average competitor could overestimate *Fagus*' equilibrium abundance. To address these limitations, future studies could extend the JAB model to include multiple species to capture these dynamics more realistically.

## 5 Conclusions

Our study largely supports the prediction of a classical theory (Ellenberg 1963) that *Fagus sylvatica* naturally predominates in Central European forests under mesic environmental conditions due to its advantageous demography compared to the average population of all *other* tree species in those environments. Towards the extremes of the water availability and pH gradient, *Fagus*' relative abundance declines. But contrary to Ellenberg's original predictions, our results do not show that *others* supplant *Fagus* at the dry margin of the soil water gradient, which could be attributed to limitations of the soil water scale used.

We further explained the environmental pattern of *Fagus*' predominance with the environmental response of demographic rates by simulating their effects on competitive equilibria. This makes our study the first to ground Ellenberg's theory in demographic mechanisms. Our demographic analysis revealed that the environmental change in *Fagus* abundance is primarily driven by variation in its own demographic rates, rather than those of the average *other* species in the respective environment. Furthermore, we identified that the environmental response of demography with the highest impact on relative basal area occurs at *Fagus*' overstory stage, even though shade‐tolerance of *Fagus* at the sapling stage is the primary factor for its predominance in the first place.

Our study presents a significant breakthrough as it demonstrates that inverse calibration of a simple forest population model with a sapling stage can be used to predict credible patterns of equilibrium abundance along environmental gradients. Moreover, this is the first study that provides a data‐driven demographic explanation for a widely assumed theory on *Fagus* abundance and predominance. This highlights the potential of inverse calibration as an alternative to directly estimating demographic rates to understand species turnover along environmental gradients. Building on this approach, future studies can predict the distributions of interacting populations and gain deeper insight into how species' dynamic interactions are shaped by their environment.

## Author Contributions

**Lukas Heiland:** conceptualization (equal), data curation (equal), formal analysis (lead), methodology (equal), software (equal), visualization (equal), writing – original draft (equal), writing – review and editing (equal). **Georges Kunstler:** conceptualization (equal), formal analysis (supporting), methodology (equal), supervision (equal), writing – review and editing (equal). **Lisa Hülsmann:** conceptualization (equal), formal analysis (supporting), funding acquisition (equal), methodology (equal), project administration (equal), supervision (lead), writing – review and editing (equal).

## Funding

This work was supported by Biodiversa+ (ANR‐20‐EBI5‐0005‐03), RESONATE REGE‐ADAPT (ANR‐24‐PEFO‐0006), Deutsche Forschungsgemeinschaft (491183248), Bayerisches Staatsministerium für Wissenschaft und Kunst, bayklif, Agence Nationale de la Recherche, ANR‐20‐CE32‐0005‐01, Open Access Publishing Fund of the University of Bayreuth.

## Conflicts of Interest

The authors declare no conflicts of interest.

## Supporting information


**Table S1:** Scope of the German NFI and plot selection.
**Table S2:** Size classes for saplings counts and corresponding radii of sampling circles in the three surveys of the German NFI (main years 1987, 2002, 2012). The size classes up to dbh 7 cm, which were consistently sampled across all three surveys, were assigned to the size class J in fitting the JAB model.
**Table S3:** Soil water levels from Benning et al. (2016), ranking and conversion into pseudo‐ratio scale. Translation according to Leuschner and Ellenberg (2017).
**Table S4:** Stratification of the two environmental gradients into seven intervals each (left: water level, top: pH) and the number of forest plots sampled within each of the 49 resulting bins.
**Table S5:** Taxa composition of the German NFI on the selected plots. All observed taxa are listed, ranked by the mean basal area per plot. In addition, the mean percentage of the total plot basal area is given.
**Table S6:** Posterior distributions of the dispersion parameter ϕ and bulk effective sample size (ESS). There are different levels of uncertainty per stage and survey.
**Table S7:** Posterior distributions of initial and equilibrium abundances (stages J and A [count ha^−1^]; B and total BA[m^2^ ha^−1^]) and populations' predominance (mean ± standard deviation across subpopulations and HMCsamples). Equilibrium states include counterfactual simulations, where the rate *s* has been switched between *Fagus* and *others* or where the respective other population does not regenerate.
**Figure S1:** The JAB model represents three different size stages of a tree population (J, A, B) and demographic processes, including the competitive interactions between size stages and multiple populations. There are processes that contribute to population growth: seedling input into the population from temporal or spatial dispersal *L*_*p*_ (dependent on regional basal area $B_{p}$), within‐population seedling regeneration *r*, transitions from a smaller to a larger stage (*g*, *h*), and the basal area growth *b*. The processes that limit population growth comprise the competition effect from the total sum of the sapling stage J on population‐specific J (*c*_*J*_), the competition from the sum of A and B, that is, the total basal area of all populations BA, on A and B (*c*_*A*_, *c*_*B*_), as well as the asymmetric competition from BA on J (“shading” effect s). The choice of thresholds between size stages is informed by the size classes in the data. In the German NFI small trees between height 20 cm and dbh 7 cm were counted, while trees with dbh > 10 cm were additionally measured in terms of basal area, so that intermediary size stage A acts as a mediator between count data for J and the basal area data for B. The factor $\beta_{\mathrm{uA}}$, that is, the upper basal area of a tree in A, converts A from counts to basal area.
**Figure S2:** Locations of the 795 randomly selected NFI clusters in Germany. Each cluster consists of one to four sampling plots, depending on whether the location was forested. One random plot per cluster was selected if its elevation was at 100–600 m, if the observation period included all of the three surveys (1987, 2002, and 2012; which excludes former EastGermany), and if therewere no records of management within the period. Each selected plot (cluster) is represented by one subpopulation in the JAB model. The map projection is Lambert Conformal Conic, the color shading indicates elevation and relief.
**Figure S3:** Environmental range of the German NFI and of *Fagus sylvatica* 's European range of distribution. To put the environmental range of the German NFI into perspective, we extracted environmental variables at all plots used in the analysis, and at locations of *Fagus sylvatica* 's presence in Europe according to The European Atlas of Forest Tree Species (de Rigo et al. 2016). As presences, we used raster cells with predicted relative probability of presence > 1% from The European Atlas of Forest Tree Species (https://forest.jrc.ec.europa.eu/en/european‐atlas/; de Rigo et al. 2016). To visualize differences in pH ranges, we used top soil pH in CaCl_2_ (European Soil Data Centre). For comparing ranges of water availability across Europe, instead of water soil level, we used a climatic water balance, described in Heiland et al. (2022). The German NFI has a narrower range with regard to both top soil pH in CaCl_2_ (A) and climatic water balance (B). Lines correspond to quartiles.
**Figure S4:** Pairs of paraboloid parameter correlations within stage J, separately for *Fagus* and *others*.
**Figure S5:** Pairs of paraboloid parameter correlations within stage A.
**Figure S6:** Pairs of paraboloid parameter correlations within stage B, separately for *Fagus* and *others*.
**Figure S7:** JAB model demographic rates in environmental space, including the density‐dependent terms at initial and equilibrium state. The contours show predictions from smoothing splines fitted to the posterior distributions (as described in Section 2.5.1).
**Figure S8:** Effect of environmentally‐responsive demography in environmental space for all rates that were determined with paraboloid parameters.
**Figure S9:** Effect of environmentally‐responsive demography in environmental space for combinations of rates.
**Figure S10:** Effect of environmentally‐responsive demography in environmental space for *L*_*p*_.

## Data Availability

Data available from the Dryad Digital Repository at https://doi.org/10.5061/dryad.3ffbg79pv (Heiland et al. 2023). All software to reproduce the study has been made openly available at https://doi.org/10.5281/zenodo.22125749.
